# Preliminary experience of CT imaging of the ischaemic brain penumbra through spectral processing of multiphasic CTA datasets

**DOI:** 10.1038/s41598-023-38370-9

**Published:** 2023-07-15

**Authors:** T. Duprez, A. Vlassenbroek, A. Peeters, P. A. Poncelet, E. Levecque, F. Austein, G. Pahn, Y. Nae, S. Abdallah, E. Coche

**Affiliations:** 1 Department of Radiology, Cliniques Universitaires Saint-Luc, Université Catholique de Louvain (UCLouvain), Avenue Hippocrate 10, 1200 Brussels, Belgium; 2 CT/AMI Clinical Science, Philips Health Systems, Avenue du Bourgmestre Etienne Demunter 1, 1090 Brussels, Belgium; 3 Department of Neurology, Stroke Unit, Cliniques Universitaires Saint-Luc, Université Catholique de Louvain (UCLouvain), Avenue Hippocrate 10, 1200 Brussels, Belgium; 4 Department of Medical Imaging, Grand Hôpital de Charleroi (GHdC), Grand’Rue, 3, 6000 Charleroi, Belgium; 5 Department of Diagnostic and Interventional Neuroradiology, University Medical Center Hamburg-Eppendorf, Martinistraße 52, 20426 Hamburg, Germany; 6 PD CT/AMI Clinical Science, Philips GmbH Market DACH, Röntgenstraße 22-24, 22335 Hamburg, Germany; 7 CT/AMI Clinical Science, Advanced Technologies Center, Philips Medical Systems Technologies Ltd., Building No. 34, P.O. Box 325, 3100202 Haifa, Israel

**Keywords:** Health care, Medical research, Neurology

## Abstract

To assess ischaemic penumbra through the post-processing of the spectral multiphasic CT Angiography (mCTA) data in acute ischaemic stroke (AIS) patients. Thirty one consecutive patients strongly suspected of severe Middle Cerebral Artery AIS presenting less than 6 h after onset of symptoms or with unknown time of onset of symptoms underwent a standardized CT protocol in spectral mode including Non Contrast CT, mCTA, and Perfusion CT (CTP) on a dual-layer MDCT system. Areas disclosing delayed enhancement on iodine density (ID) maps were highlighted by subtraction of the serial mCTA datasets. Two neuroradiologists independently rated the correspondence between delayed enhancing areas at mCTA and the penumbral/infarcted areas delineated by two validated CTP applications using a 5-levels scoring scale. Interobserver agreement between observers was evaluated by kappa statistics. Dose delivery was recorded for each acquisition. Averaged correspondence score between penumbra delineation using subtracted mCTA-derived ID maps and CTP ones was 2.76 for one application and 2.9 for the other with best interobserver agreement kappa value at 0.59. All 6 stroke mimics out of the 31 patients’ cohort were correctly identified. Average dose delivery was 7.55 mSv for the whole procedure of which CTP accounted for 39.7%. Post-processing of spectral mCTA data could allow clinically relevant assessment of the presence or absence of ischaemic penumbra in AIS-suspected patients if results of this proof-of-concept study should be confirmed in larger patients’series.

## Introduction

Imaging work-up of acute ischaemic stroke is challenging, necessitating the need for efficient treatment strategies^1,2^. Patients’ triage between true strokes and stroke mimics and accurate delineation of the salvageable ischaemic penumbra are the two main goals when the patient is admitted. The penumbral imaging has nowadays migrated from the Diffusion-Weighted Imaging/Perfusion-Weighted Imaging (DWI/PWI) mismatch at MRI to the CT scanner Cerebral Blood Volume/Relative Mean Transit Time (CBV/rMTT)^3^ or Time To Maximum/Relative Cerebral Blood Flow (Tmax/rCBF)^4–6^ mismatch because of the need to reduce the door-to-needle time. The standard CT work-up includes the NCCT, CTA, and CTP in most cases. Penumbral imaging CTP appears increasingly mandatory as the demonstration of the presence of salvageable penumbra in eloquent brain area allows extension of the therapeutic frame of both thrombolysis and thrombectomy^7–9^. In addition, a growing corpus of clinical studies have demonstrated better outcomes after thrombectomy than after thrombolysis^10,11^. Penumbral imaging has become decisive for thrombectomy in patients presenting with a significant delay from last time seen well in whom mCTA reveals both a large vessel occlusion (LVO) and with bad collaterals^12,13^. In other cases, a negative CTP reinforces the conviction for a non ischaemic process in clinically inconclusive cases. An alternative mCTA-derived imaging of the penumbra avoiding the irradiating CTP and sparing the second bolus injection of iodinated contrast medium (CM) for CTP could be clinically relevant.

Conventional CT scanners use a detector technology which has the crucial disadvantage that the detected X-ray photons are integrated, and thus unable to extract the spectral information of the polychromatic X-ray spectrum passing through the imaged object. In contrast, current clinical Spectral CT scanners (also refered to as Dual-Energy CT scanners) enable the discrimination between different materials based on the differential X-ray attenuation properties in two “energy bands” of the spectrum instead of averaging the entire polychromatic X-ray beam. In other words, the spectral dependencies of the net X-ray attenuation can be imaged and analyzed as a material characteristic and can be used to discriminate tissues beyond the Hounsfield Unit (HU) paradigm. With this technology, material concentration maps can be obtained using two or three-material decomposition algorithms^14^. In vascular imaging, iodine density (ID) images, also referred to as iodine maps, are obtained from an iodine-water decomposition and scaled to the true iodine concentration in units of mg/ml. Voxels without iodine have zero iodine and hence are black.

We hypothesized that an easy-to-perform post-processing of triphasic CTA-derived ID spectral images could provide an accurate assessment of the presence of ischaemic penumbra, and even a valuable estimate of its extent, thereby opening up a potential field for CT imaging of penumbra without the need to perform CTP.

## Material and methods

### Patients’ population and inclusion criteria

Thirty-one consecutive patients (18 female and 13 male, average age 77 years) presenting with a strong suspicion of severe MCA stroke less than 6 hours after onset of symptoms (n = 24) or unknown time of onset (n = 7) were included into the study.

Inclusion criteria were: combination of severe right hemiparesis and aphasia or combination of severe left hemiparesis and extinction/inattention.

Exclusion criteria were: high probability of stroke mimic (history of chronic refractory epilepsy); high clinical suspicion for seizure; known unequilibrated diabetes.

### CT scan protocol

All patients underwent brain mCTA and CTP after hemorrhage had been excluded by initial NCCT. A dual-layer spectral detector MDCT scanner (IQon^®^, Philips Healthcare^®^, Cleveland, OH, USA) was used. Initial CTA run (t_1_) was triggered by bolus tracking at maximal arterial enhancement after IV injection of an average of 65 cc (range 64.7–90) of iodinated contrast agent (Xenetix 350, Guerbet, Paris, France) using a power injector. The 3D X-Ray dose modulation option was activated and the acquisition covered the neck and the head with an average scan time of 3.2 s (range 2.7–3.6) and an average scan length of 34.1 cm (range 27.6–39.1). The average injection rate was 4.8 cc/s (range 2.9–5.8). The second (t_2_) and third (t_3_) CTA runs covered only the brain without the dose modulation option after a delay of 7 and 20 s after the t_1_ run respectively, with a scan time of 2.3 s, and a scan coverage of 8 cm. All mCTA scans were acquired in spiral mode with minimal interscan delay, at 120 kVp and at an average of 92 mAs (range 58–174) for t_1_, and of 190 mAs for t_2_ and t_3_ phases. ID spectral images of the mCTA were reconstructed with a slice thickness of 0.9 mm. CTP was acquired with a 64 × 0.625 mm collimation (4 cm) in a so-called dynamic axial ‘Jog Mode’ resulting in a total brain coverage of 8 cm with 40 phases acquired every 3.6 s after power injection of 52 cc of contrast media (CM). All CTP images were acquired at 120 kVp and 52 mAs and CTP conventional images were reconstructed with a slice thickness of 5 mm.

### CT data post-processing

#### Conventional CTP post-processing

Two commercially available post-processing applications were used to analyze the conventional CTP data.

**Figure 1:**
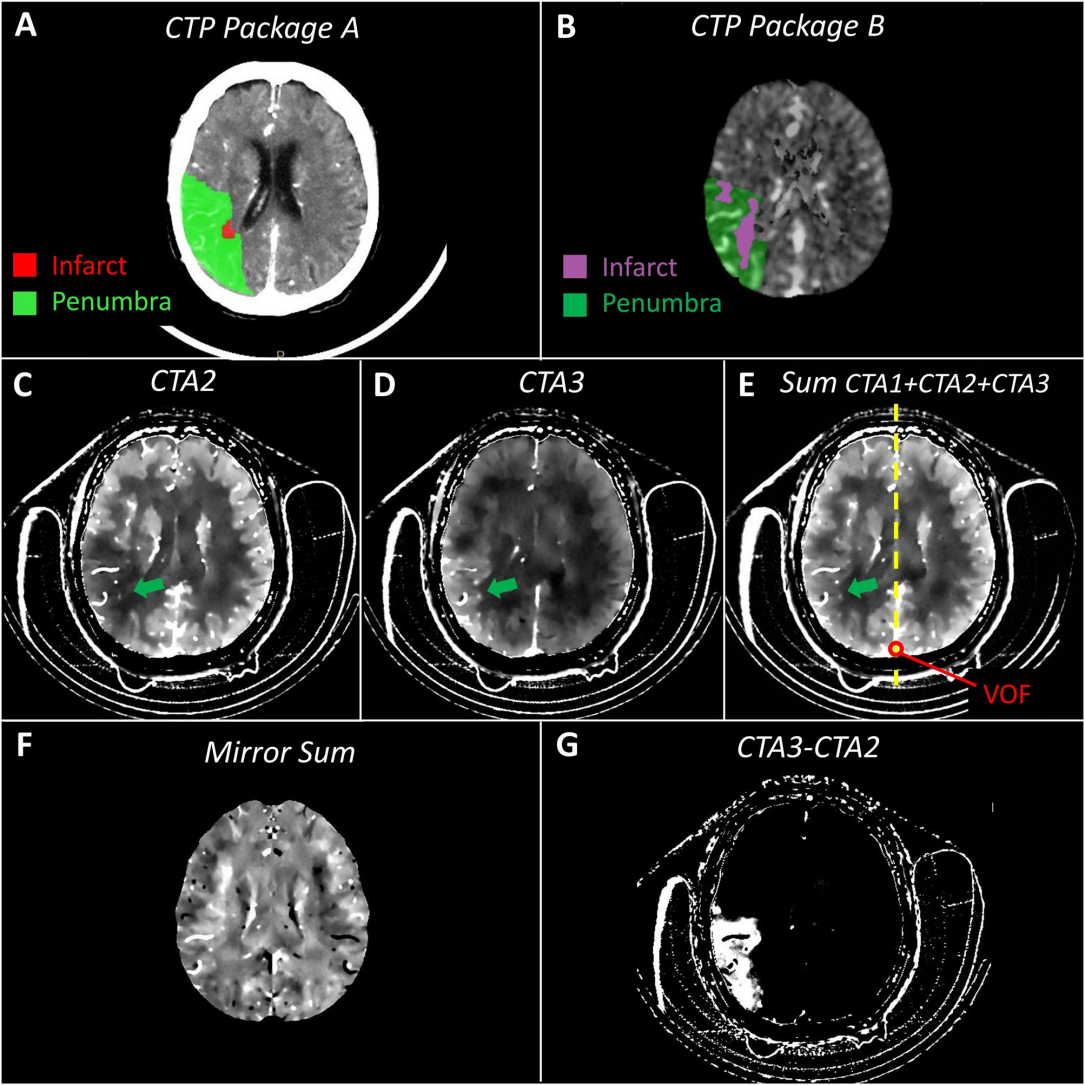
Overview of displayed mapped images in similar slice location in a ischaemic patient with penumbra. (**A**) Summary maps obtained with the post-processing of conventional CTP data with Philips Intellispace^®^ software (Package A) (**B**) Summary maps obtained with the post-processing of conventional CTP data with RAPID^®^ software (Package B). (**C**) Spectral ID map obtained from CTA acquisition at time t_2_: hypoperfused area arrowed in green. (**D**) Spectral ID map obtained from CTA acquisition at time t_3_: delayed enhancement of the territory being hypoperfused at t_2_ (previous illustration), ID maps at t_2_ and t_3_ being displayed with the same window settings. (**E**) Sum of the spectral ID maps obtained with all CTA acquisitions, the Venous Output Function (VOF at three time points) being measured in the superior sagittal sinus vein (red ROI) and used to normalize the ID maps at each time point. The dashed yellow line represents the symmetry axis between the two hemispheres. (**F**) Mirror image highlighting side-to-side asymmetry of the sum image (previous illustration) using the symmetry axis between the two hemispheres. The ratio between the ID content of each voxel on one side of the brain with the corresponding mirrored one of the contralateral side is displayed. (**G**) Positive differences between the normalized ID maps at t_3_ and t_2,_ highlighting the area with delayed enhancement. *Note* at this slice location, the average correspondence scores between (**G**) and (**A**, **B**) were (3.5, 3) for the penumbra; was zero between (**F**) and (**A**) for the infarct and finally were 3.5 and 2 between (**A**) and (**B**) for the penumbra and the infarct respectively.

*Application A:* CTP infarct/penumbra summary maps were obtained using the standard processing provided by the manufacturer's software (IntelliSpace Portal 10^®^, Philips Healthcare^®^). The software used a delay-sensitive algorithm. The rMTT and CBV distinguished the total ischaemic penumbra and the infarcted core with standard thresholds set at rMTT > 150% for penumbra when compared to the contralateral hemisphere, and at CBV value < 2.0 mL/100 g for infarct (Fig. 1A).

*Application B:* Another commercially application (RAPID^®^ (iSchemaView Inc^®^, Menlo Park, CA, USA)) was used that employed a delay-insensitive algorithm. Standard thresholding was applied to assess the total hypoperfused area with a Tmax delay > 6 s and the infarcted core with a rCBF value < 30% (Fig. 1B).

#### Spectral mCTA post-processing (Fig. 2)

**Figure 2:**
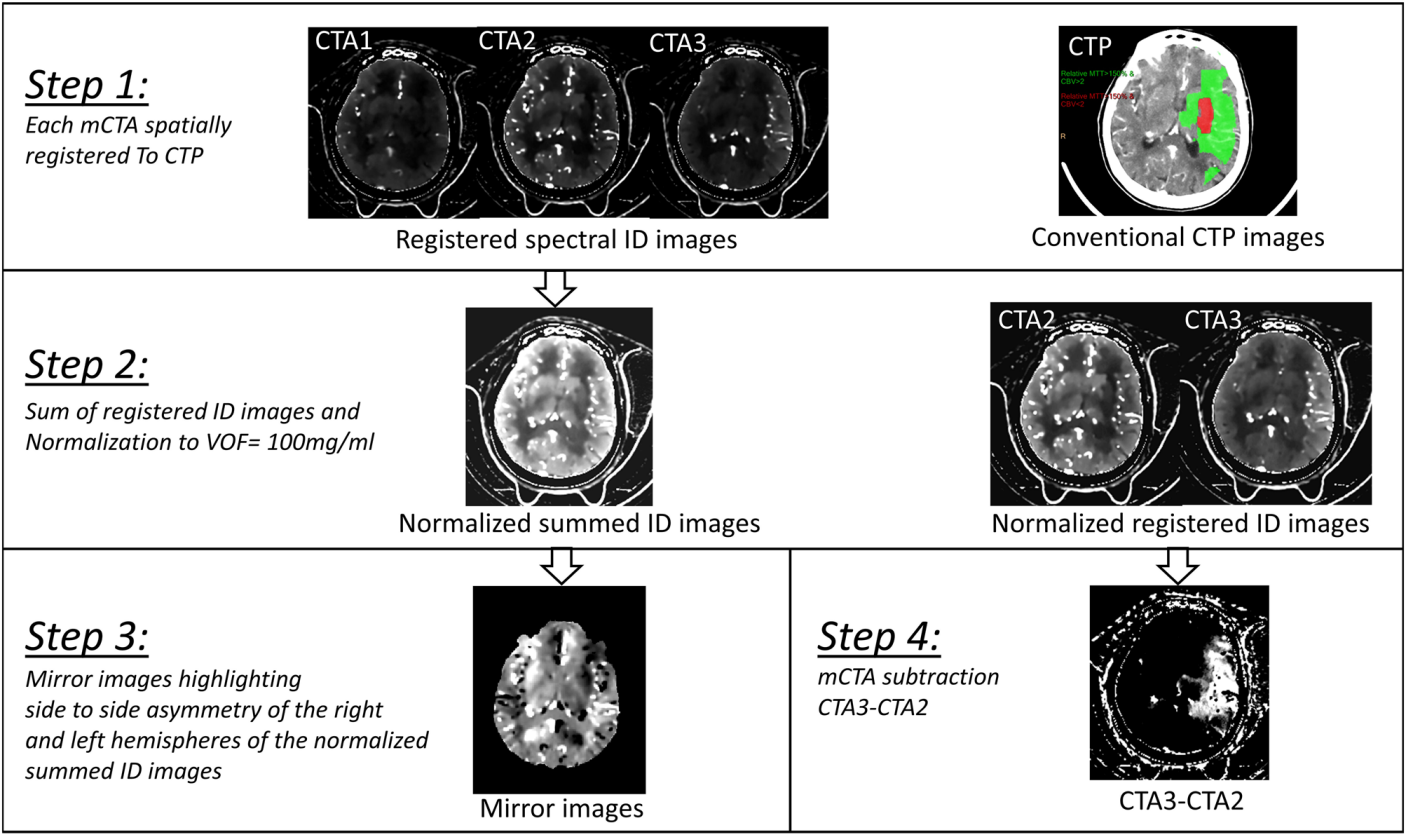
Summary of the spectral mCTA post-processing workflow. Step 1: ID spectral images of the mCTA were registered to the CTP dataset using an elastic registration algorithm generating registered datasets at 5-mm-thick slices; Step 2: A sum of the 3 registered ID mCTA datasets was calculated. A region of interest (ROI) was drawn in the superior sagittal sinus vein of the summed ID images to normalize the venous output function (VOF) to 100 mg/ml. The normalization factor was then applied to the registered ID mCTA datasets; Step 3: iodine concentration values from the normalized summed ID images were converted into relative differences, to highlight side-to-side asymmetry; Step 4: brain areas disclosing delayed enhancement on the registered normalized ID maps were highlighted by subtracting the t_2_ phase dataset from that of the last t_3_ phase. All voxels displaying negative values in the corresponding subtracted ID images were clamped to zero.

In a first step (Step 1 in Fig. 2), ID spectral images of the mCTA were registered to the CTP dataset using an elastic registration algorithm (FEIR^®^, Philips Research Hamburg, Germany), generating registered datasets at 5 mm-thick slices^15^ (Fig. 1C,D). All additional calculations on the registered ID datasets were performed using the ImageJ software package (ImageJ 1.52a, National Institutes of Health, Bethesda, MA, USA) and proprietary coding using C++ and C# which are standard programming languages (Visual Studio 2010^®^, Microsoft^®^ Corporation, Redmond, WA, USA).

In Step 2, a sum of the 3 registered ID mCTA datasets was calculated. A region of interest (ROI) was drawn in the superior sagittal sinus vein of the summed ID images and the iodine concentration (IC) measured, mimicking (with only three time points) the venous output function (VOF) which is typically used to normalize CTP data to minimize inter-patient variability^16^ (Fig. 1E). The original registered ID datasets of the mCTA were then multiplied by a factor 100/IC to normalize the total iodine concentration in the venous outflow of each patient to a value of 100 mg/ml.

In Step 3, iodine concentration values from the normalized summed ID images were converted into relative differences, to highlight side-to-side asymmetry. The conversion was performed comparing each voxel on one side of the brain with the mirror voxel in the contralateral hemisphere. This comparison used the symmetry axis of the skull to separate the right and left hemispheres. The degree of relative difference was quantified as the ratio (expressed as a percentage) between the ID content of each voxel on one side of the brain with the corresponding mirrored one of the contralateral side. The method generated mirror images displaying hypoperfused areas as darker voxels (ratio < 100%) compared to the brighter voxels (ratio > 100%) in the mirrored area on the contralateral side (Fig. 1F).

In Step 4, brain areas disclosing delayed enhancement on the registered normalized ID maps were highlighted by subtracting the t_2_ phase dataset from that of the last t_3_ phase. All voxels displaying negative values in the corresponding subtracted ID images were clamped to zero (Fig. 1G).

A summary of the spectral mCTA post-processing workflow is described in Fig. 2.

### Observers’ evaluation technique and scoring system

A standardized reading grid was prepared for each patient displaying the ID summed images, the mirror summed images, the subtracted maps and the CTP summary maps from the 2 CTP commercial applications at 3 slice locations displaying the largest hypoperfused area. The windowing to display the mirror sum images (window level = 100%, window width = 60%) and the subtracted spectral images (window level = 0.5 mg/ml, window width = 0.5 mg/ml) was the same for all patients. Two trained neuroradiologists (Reader 1 and Reader 2) blinded to patients’ history, independently rated by visual examination the correspondences between: (i) the hypoperfused areas on the mirror summed images and the infarcted area obtained by Application A (Step I); (ii): the delayed enhancing areas visible in the subtracted maps and standard CTP penumbra obtained using the 2 applications (Step II); (iii): the infarct and the penumbra mapped from CTP data using the 2 applications (Step III). The observers used a 5-point scale (0 = no correspondence; 1 = low correspondence; 2 = intermediate correspondence, 3 = good correspondence, 4 = excellent correspondence). Repeatability of the ratings was assessed by a second reading (Step II) with the software A 6 months later.

### Statistical testing

The reproducibility/repeatability and the strength of agreement between readers was evaluated by linear kappa statistics.

### Ethical issues

The study was carried out after clearance of the study protocol by the ethics committee of the Cliniques Universitaires Saint-Luc of the Université Catholique de Louvain (EC visa: UCLouvain 2017/12AVR/209. All methods were performed in accordance with the relevant guidelines and regulations and informed consent was obtained from all subjects and/or their legal guardian(s). The study protocol was first registered on 14/05/2019 as N° NCT03948425 on Clinicaltrials.gov database.

## Results

The relevant clinical data of the enrolled patients, including delay, site of occlusion, volumes of infarcted and penumbral areas are given in Table 1 together with treatment and modified Rankin’s score (mRS) at 3 months.

**Table 1:** Patients’ data.

#	Age	Sex	Delay/LSW*	Site of occlusion	PV RAPID	CV RAPID	PV Philips	CV Philips	Vol INFARCT 24 h	Treatment	mRS 3 m	Cause of death	Mimic
1	51	F	00:23	NO STOP	0	0	34	1	0 (DWI)	glucose	0		Hypoglycemia
2	82	F	01:11	LEFT ICA	345	116	56	193	NA (HT on CT)	IVT + TBY	6	PH2 + IVH	
3	62	F	00:58	RIGHT MCA (M1)	84	63	130	39	0 (CT)	IVT + TBY	2		
4	94	F	17:26*	LEFT CCA + ICA + MCA	123	268	50	213	NA (no CT nor MR)	SUC	6	Stroke (BSC)	
5	88	F	02:03	RIGHT MCA (M2)	65	15	104	12	< 1 ml (DWI)	IVT + TBY	0		
6	90	F	01:58	RIGHT MCA (M1)	170	4	188	42	0 (CT)	TBY	4		
7	74	M	01:02	LEFT MCA (M1)	73	0	176	4	< 1 ml (DWI)	IVT + TBY	0		
8	84	M	02:36	RIGHT PCA (P2)	140	24	45	36	24 ml (DWI)	IVT	4		
9	58	M	02:48	LEFT ICA + MCA	103	160	156	158	173 ml (DWI)	IVT + TBY + DH	4		
10	86	F	16:02*	RIGHT CCA + ICA	87	0	212	8	10 ml (CT)	CEA	6	Stroke (BSC)	
11	94	F	05:19	NO STOP	19	0	48	3	NA (HT on CT)	IVT	6	Stroke (BSC)	
12	75	F	01:06	LEFT ICA	143	17	150	29	14 ml (DWI)	IVT + TBY	1		
13	67	M	05:24	RIGHT MCA (M1)	71	0	40	10	8 ml (DWI)	IVT	0		
14	72	F	01:55	NO STOP	7	0	4	3	0 (DWI)	IVT	0		
15	85	M	02:20	NO STOP	0	0	6	2	0 (DWI)	SUC	2		RDAS
16	83	M	01:36	LEFT ICA + MCA + ACA	187	231	85	263	NA (no CT nor MRI)	SUC	6	Stroke (BSC)	
17	77	M	00:51	RIGHT MCA (M2)	136	43	169	35	11 ml (CT)	IVT + TBY	0		
18	57	M	01:55	PCA (P2)	157	0	50	13	20 ml (DWI)	IVT	1		
19	90	F	12:39*	LEFT MCA (M1)	151	128	27	131	122 ml (CT)	IVT	6	Stroke (BSC)	
20	63	M	01:06	LEFT MCA (M1)	30	57	35	67	92 ml (CT)	IVT	1		
21	86	M	09:01*	RIGHT ICA	148	0	243	12	7 ml (DWI)	TBY	3		
22	56	M	04:39	NO STOP	12	0	153	20	0 (DWI)	SUC	3		Psychosomatic?
23	73	M	11:20*	RIGHT MCA (M2)	35	0	43	8	5 ml (DWI)	IVT + TBY	2		
24	78	M	01:19	RIGHT ICA	74	0	33	8	3 ml (CT)	CEA	4		
25	89	F	03:07	NO STOP	3	0	23	5	0 (DWI)	SUC	3		Migraine
26	79	F	02:24	NO STOP	19	9	152	19	0 (DWI)	IVT	2		
27	93	F	09:12*	RIGHT MCA (M1)	71	0	71	1	NA (no CT nor MR)	SUC	6	Stroke (BSC)	
28	58	F	02:22	NO STOP	4	0	13	9	NA (no CT nor MR)	SUC	0		Migraine
29	87	M	04:44	NO STOP	0	0	28	13	11 ml (CT)	SUC	5		
30	90	F	03:13	NO STOP	0	0	17	1	5 ml (DWI)	IVT	6	Vascular death	
31	55	F	09:11*	NO STOP	3	0	59	6	NA (no CT nor MR)	SUC	2		PRES

*PV* penumbra volume; *CV* core volume; *mRS 3 m* modified Rankin’s score at 3 months; *LSW* last seen well; *HT* hemorrhagic transformation; *IVT* intravenous thrombolysis; *TBY* thrombectomy; *SUC* stroke unit care; *DH* decompressive hemicraniectomy; *CEA* carotid endarterectomy; *BSC* best supportive care, *PH2* parenchymal hemorrhage type 2; *IVH* intraventricular hemorrhage; *PRES* posterior reversible syndrome; *RDAS* recurrence of deficits after stroke.

The average normalization factor to 100 mg/ml of IC was 3.62 (range 2.39–5.56) with a corresponding measured IC in the sagittal sinus on the summed images ranging from 17.97 to 41.90 mg/ml.

Table 2 gives the average score (with the standard deviation between parentheses) per reader and the average correspondence between readers for the thirty one patients. The reproducibility and strength of agreement between the readers was given by the kappa value. Step I: average score = 1.76 (Reader 1: 2.41; Reader 2: 1.12) with a fair agreement between readers (kappa = 0.29); Step II: average scores = 2.76 (Reader 1: 2.91; Reader 2: 2.61) and 2.9 (Reader 1: 3.03; Reader 2: 2.76) with moderate agreement between readers (kappa = 0.59 and 0.59) for software applications A and B respectively and Step III: average scores = 2.68 (Reader 1: 2.63; Reader 2: 2.73) and 3.05 (Reader 1: 2.98; Reader 2: 3.13) with good and very good agreements between readers (kappa = 0.84 and 0.69) for penumbra and infarct respectively.

**Table 2:** Average correspondence score (with standard deviation between parenthesis) per and between readers for the 31 patients and K agreement coefficient.

Penumbra	Reader 1	Reader 2	Average	Kappa
Spectral substraction-Package A (reading 1)	2.91 (1.18)	2.61 (1.2)	2.76	0.59
Spectral substraction-Package A (reading 2)	2.89 (1.13)	2.58 (1.38)	2.74	0.63–0.7
Spectral substraction-Package B	3.03 (1.12)	2.76 (1.18)	2.9	0.59
Package A–Package B	2.63 (1.19)	2.73 (1.22)	2.68	0.69
Infarct				
Spectral Mirror Sum-Package A	2.41 (1.58)	1.12 (1.55)	1.76	0.29
Package A–Package B	2.98 (1.68)	3.13 (1.42)	3.05	0.84

Scoring system: 0 = no correspondence; 1 = low correspondence; 2 = intermediate correspondence; 3 = good correspondence, 4 = excellent correspondence.

The second reading for Step II after a wash-out period of 6 months gave an average score of 2.74 (Reader 1: 2.89; Reader 2: 2.58) with a good repeatability for both readers (kappa Reader 1: 0.63 and kappa Reader 2: 0.7).

**Figure 3:**
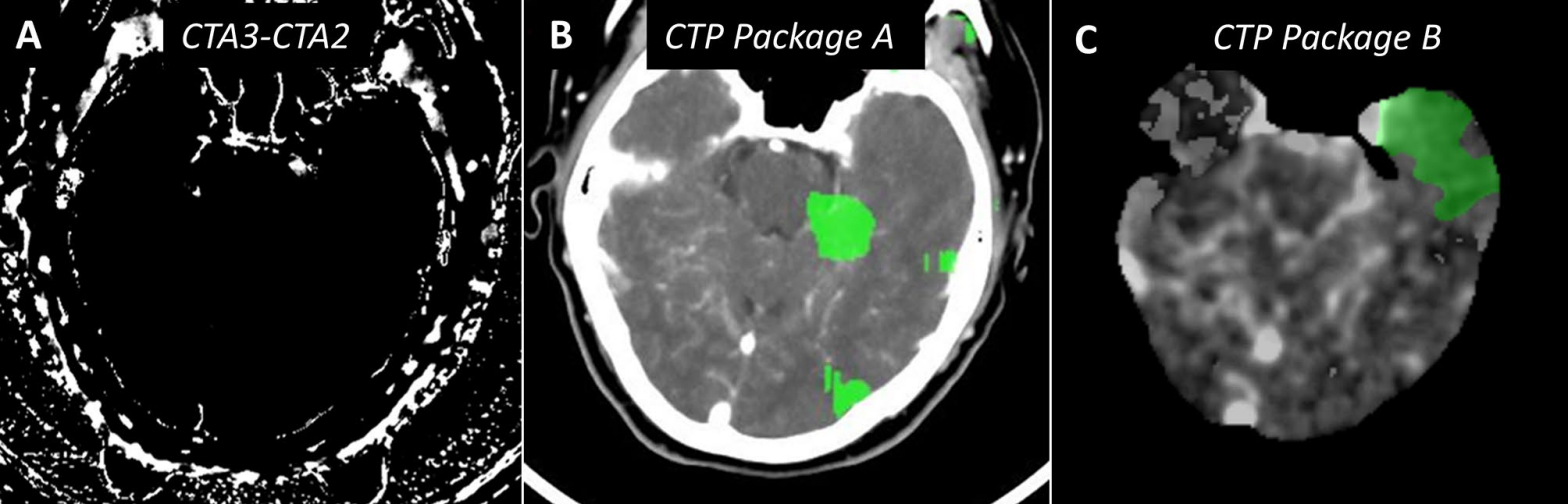
Stroke mimic patient (hypoglycemia): summarized work-up. All slices in similar slice location. (**A**) negative subtracted ID map between t_3_ and t_2_ (**B**) false positive penumbra using the package A (Philips Intellispace^®^) (**C**) false positive penumbra using the package B (RAPID^®^).

Among the entire patients’ cohort, six stroke mimics were identified and confirmed at clinical work-up. Causes of stroke-like symptoms in those patients were hypoglycemia, seizure on ischaemic cortical scar, psychosomatic disorder, migraine and Posterior Reversible Encephalopathy Syndrome (PRES) in one patient treated by tacrolimus. None of them had delayed enhancement on the subtracted spectral iodine maps but five of them had at least one false positive penumbra area on the CTP summary maps of the 3 slices presented for scoring. Four false positive patients were identified with software Application A and three with Application B, thereby lowering the scores of correspondence between mCTA subtracted spectral images and infarct/penumbra CTP maps obtained with each application. Two patients had false positive penumbral areas with both applications (Fig. 3).

The average Dose Length Product (DLP) (head phantom16) was 754 mGy.cm, 1441 mGy.cm and 1424 mGy.cm for NCCT, mCTA and CTP respectively. The total effective X-ray dose of the CT procedure was 7.55 mSv (k = 0.0021)^17^. The CTP represented 39.7% of the average X-ray dose and 44.4% of the average contrast medium dose of the entire CT procedure.

## Discussion

The added value of the spectral CT in acute stroke work-up has already been demonstrated as it allows improved detection of infarcted brain tissue, parenchymal edema mapping, and vessel occlusion localization^18–23^. Recent literature has highlighted the value of the collaterals evaluation in order to extend the delay between symptoms onset and thrombectomy^24^. At 24 h post-treatment follow-up NCCT, spectral CT has shown the capability to distinguish between extravasated hyperdense blood featuring the true hemorrhagic transformation (HT) and extravasated CM featuring the ‘iodinated pseudo HT′^25^. Our investigation of penumbra imaging without CTP shows a significant benefit of spectral CT in hyperacute ischaemic stroke work-up. The accuracy of this method does not yet compete with that of CTP, with the reserve that both significance and accuracy of CTP-defined penumbra still remain debated and are significantly dependent upon parametrization and processing algorithms^26–28^. To minimize over/underestimation of the penumbra, most standard thresholds were applied to both applications, for example rCBV < 2 ml/100 g and rMTT > 150% for Application A and Tmax < 6 s and rCBF < 30% for Application B. Our results show that the correspondence scores for penumbra delineation between two clinically validated CTP post-processing applications using different algorithms and parameters were inferior to the correspondence results between each of them and the penumbra as evaluated by our method (Table 2). This suggests that a sufficiently accurate assessment of the ischemic penumbra was enabled by the spectral processing of triphasic CTA data and could therefore open the field for CT imaging of penumbra without CTP, thereby reducing both the radiation and iodinated CM delivery. The mCTA modality was proposed by authors for LVO detection and for quality assessment of collaterals allowing extension of the delay between symptoms onset and the revascularization treatment^24,29,30^. The spectral analysis of the mCTA proposed here is a promising low hanging fruit approaching penumbral imaging as it does not require additional scans but only a batch-filed background post-processing of data being acquired anyway. The post-processing was performed in about 4 min which was insufficient to allow for an appropriately timed decision to not perform CTP. Automation of successive algorithmic calculations could yield results in less than one minute thereby allowing decisions in the day-to-day clinical setting.

**Figure 4:**
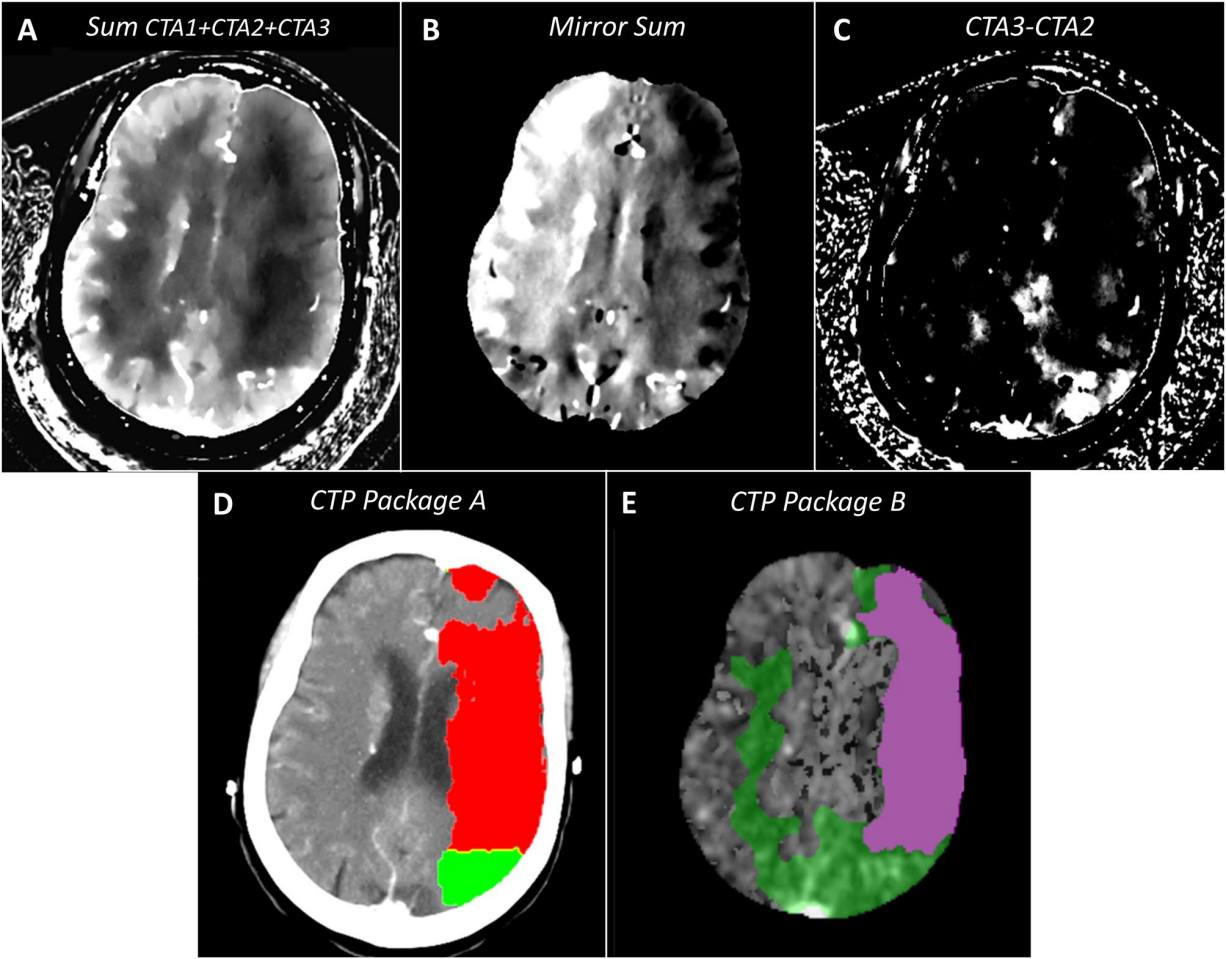
Stroke patient with prominent infarcted core and precise delineation of small penumbral area. All views in similar slice location. (**A**) sum of the spectral ID maps obtained with all CTA acquisitions. (**B**) mirror sum image (**C**) spectral CTA subtracted summary map showing the positive difference between the normalized ID maps at t_3_ and t_2_. (**D**) conventional CTP summary map using package A (Philips Intellispace^®^) (**E**) conventional CTP summary map using package B (RAPID^®^). *Note* at this slice location, the average correspondence scores between (**C**) and (**D**, **E**) were (3.5, 3) for the penumbra; was 3.5 between (**B**) and (**D**) for the infarct and finally were 3 and 4 between (**D**) and (**E**) for the penumbra and the infarct respectively.

One of the challenges of our method was the poorly accurate delineation of the infarcted core in most cases as the additional post-processing using mirrored summed images for core delineation at three time points resulted only in minor correspondence scores with CTP-based core delineation. In turn, the assessment of the penumbra requires only the acquisition of the mCTA ID maps at two time points, one which maximizes the enhancement of the healthy tissues and one at a later time which maximizes the enhancement of the ischaemic tissues. But better infarct delineation could be reached by appropriate thresholding of the accurate CBV mapped images. The CBV is measured as the area under the time attenuation curve and above the baseline in the conventional CTP images or equivalently as the integration of the ID values over all time points in spectral CTP images^31^. For patients where there was still a residual but limited CBF, the sum of the mCTA iodine densities at the three time points was therefore not an accurate approximation of the total area under the curve which we can get from dynamic CTP with more sampling points (see Fig. 1F). However in a few patients, our post-processing using mirrored analysis allowed a valuable assessment of the core, and showed a good correspondence with CTP whenever there was a very severe CBV reduction (and thus a severe CBF reduction) in cortical areas (Fig. 4). Future technological improvements allowing more sampling points could result in enhanced accuracy of infarcted core delineation using our proposed technique.

It must be highlighted that none of the six stroke mimics had late iodine enhancement on spectral subtraction maps, suggesting a strong negative predictive value (NPV) of the method. Of note, five of them had false positive CTP results using the two application packages, thereby reinforcing the presumption of high NPV of the subtracted maps when compared to CTP ones (Fig. 3). This observation was obtained only on a small patients’ cohort and would therefore require further validation on larger groups. If confirmed, the high NPV could have a significant breakthrough impact in the daily practice as CTP is frequently performed not only for therapeutic decision but also for diagnosis of ischaemic versus non ischaemic event. In such cases in whom CTP is performed for diagnostic exclusion of brain hypoperfusion, on-line real time assessment of negative processing of spectral mCTA could prevent the full NCCT/CTA/CTP cascade thereby avoiding drawbacks of unwarranted CTP acquisitions and/or avoiding the need for performing a secondary diffusion-weighted sequence on busy MRI centers.

Further technical developments will be needed to enable this on-line real-time automatic post-processing of spectral results at the imaging console to render it clinically efficient and viable.

Our study had significant limitations. It was mainly a feasibility and proof-of-concept study aimed at opening a potential investigational field for the routine practice in the field of acute ischaemic stroke.

Accordingly, the study cohort was small based on the recommendations of the EC of the institution. The recruitment was restrictively limited to clinically severe strokes presumptively due to proximal MCA occlusion thereby inducing a bias when compared to the day-to-day practice in which full NCCT/CTA and,—if necessary—CTP cascade is performed in all suspected acute stroke patients. Consequently, recruitment of more appropriate prospective cohorts are warranted to validate the use of this technique in a routine situation, but significant recruitment bias of this proof-of-concept study design did not allow for a robust calculation of the sample size required for a validation study design.

## Conclusion

The evaluation of AIS patients using CT typically requires serial scans: NCCT to exclude haemorrhage, CTA to detect LVO, and CTP to assess penumbra. Aside from the specific benefits of spectral CT for better delineation of infarcted tissue and clot delineation on CTA images, we highlighted its potential to detect and approximate penumbra through a post-processing of the multiphase CTA datasets. If the results of this preliminary proof-of-concept study is confirmed in larger patients cohorts, optimization of acquisition parameters and implementation of a fast processing on-line real-time software could allow the interruption of full NCCT/CTA/CTP cascade.

## Data Availability

The datasets analysed in the current study are available from the corresponding author upon reasonable request.

## Abbreviations

CBF: Cerebal blood flow
CBV: Cerebral blood volume
CM: Contrast medium
CTA: Computed tomography angiography
CTP: Computed tomography perfusion
DWI: Diffusion-weighted imaging
HT: Hemorrhagic transformation
IC: Iodine concentration
ID: Iodine density
LVO: Large vessel occlusion
MCA: Middle cerebral artery
mCTA: Multiphasic CTA (3 phases in our institution)
mRS: Modified rankin’s score
NCCT: Non contrast computed tomography
PWI: Perfusion-weighted imaging
rCBF: Relative cerebal blood flow
rMTT: Relative mean transit time
Tmax: Time to maximum of the residue function
VOF: Venous output function
